# Bidirectional interdomain crosstalk in a *Porphyromonas gingivalis* chimeric enzyme coordinates catalytic synergy for aromatic amino acid biosynthesis

**DOI:** 10.3389/fmicb.2025.1601098

**Published:** 2025-06-13

**Authors:** Yiyan Yu, Jing An, Yu Bai, Qinghua Xu

**Affiliations:** ^1^Anhui Academy of Medical Sciences, Anhui Medical College, Hefei, China; ^2^School of Stomatology, Anhui Medical University, Hefei, China; ^3^Anhui Provincial Center for Disease Control and Prevention, Hefei, China

**Keywords:** DAH7PS-CM, *Porphyromonas gingivalis*, bifunctional enzyme, heterodomain interface, polar contacts, interdomain communication

## Abstract

The shikimate pathway, critical for bacterial aromatic amino acid biosynthesis, represents a prime therapeutic target due to its absence in humans. This study elucidates the structural and functional interplay within the bifunctional enzyme DAH7PS-CM from *Porphyromonas gingivalis* (*Pgi*DAH7PS-CM), a keystone periodontal pathogen. Integrating AlphaFold3-predicted models with biochemical validation, we identified two interdomain interfaces: a conserved DAH7PS dimerization interface and a polar interaction-driven D-CM interface (e.g., E287/R291). Mutagenesis of these residues and exposure to high Na^+^ concentrations disrupted enzyme function, confirming polar networks mediate domain crosstalk. The DAH7PS domain’s dimerization relies on conserved interfaces homologous to monofunctional DAH7PS enzymes, while the CM dimer substitutes structural roles through distinct interfacial features. Phylogenetic analysis indicates DAH7PS-CM’s specificity to periodontal pathogens, suggesting adaptive selection for domain fusion to synchronize catalytic steps. Our findings highlight the D-CM interface as a nexus for quaternary stability and allosteric communication, enabling coordinated pathway flux. These insights provide a structural basis for targeting interfacial networks with salt-modulating inhibitors or engineered disruptors, offering novel strategies to impede bacterial virulence and biofilm-associated infections.

## 1 Introduction

The shikimate pathway is a central metabolic route responsible for the biosynthesis of aromatic amino acids (phenylalanine, tyrosine, and tryptophan) and a multitude of secondary metabolites critical for bacterial survival, virulence, and biofilm formation (Herrmann and Weaver, 1999). Unlike mammals, bacteria rely exclusively on this pathway to synthesize these essential compounds, making it a prime target for antimicrobial strategies (Jensen et al., 1989; Walsh et al., 1990; Herrmann and Weaver, 1999). Metabolomic and proteomic studies have further highlighted the pathway’s upregulated activity during early bacterial biofilm development, underscoring its pivotal role in microbial colonization and pathogenesis (Yeh et al., 2020; Liu et al., 2022). Among the enzymes governing this pathway, 3-deoxy-D-*arabino* heptulosonate-7-phosphate synthase (DAH7PS) and chorismate mutase (CM) occupy key regulatory and catalytic roles, directing carbon flux and ensuring metabolic efficiency through allosteric feedback mechanisms (Herrmann and Weaver, 1999; Kaleta et al., 2013; Jiao et al., 2020).

DAH7PS catalyzes the first committed step of the shikimate pathway, condensing D-erythrose-4-phosphate (E4P) and phosphoenolpyruvate (PEP) into 3-deoxy-D-*arabino* heptulosonate-7-phosphate (DAH7P) (Herrmann and Weaver, 1999). This product undergoes six enzymatic transformations to yield chorismate, a versatile precursor for aromatic amino acids and other vital metabolites, including salicylic acid, folic acid, vitamin K2, and ubiquinone (Jensen et al., 1989; Walsh et al., 1990; Herrmann and Weaver, 1999). CM then channels chorismate into prephenate for phenylalanine and tyrosine synthesis or into anthranilate for tryptophan production (Herrmann and Weaver, 1999). Beyond catalysis, DAH7PS and CM act as metabolic checkpoints, dynamically regulated by feedback inhibition from pathway intermediates (e.g., prephenate) and end products (e.g., phenylalanine, tyrosine, tryptophan) to prevent wasteful overproduction (Jiao et al., 2020). Such regulation highlights their dual roles as catalysts and gatekeepers of metabolic flux.

**FIGURE 1:**
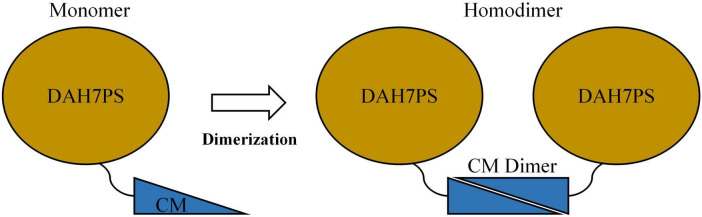
Schematics of structural organization of either monomeric or homodimeric *Pni*DAH7PS-CM.

Recent studies have identified a unique subclass of bifunctional chimeric enzymes, DAH7PS-CM, encoded by a single gene and predominantly found in periodontal pathogens of the *Prevotella* and *Porphyromonas* genera (Wu and Woodard, 2006; Finn et al., 2013; Bai et al., 2019). These enzymes integrate DAH7PS and CM activities into a single polypeptide chain, forming an N-terminal DAH7PS domain and a C-terminal CM domain connected by a short flexible linker (Figure 1; Wu and Woodard, 2006; Bai et al., 2019). Structural analysis of DAH7PS-CM from *Prevotella nigrescens* (*Pni*DAH7PS-CM) revealed that the two domains fold independently yet rely on dimerization via the CM domains for full catalytic activity (Figure 1; Bai et al., 2019). Remarkably, separating the DAH7PS and CM domains of *Pni*DAH7PS-CM by disrupting the linker abolishes nearly all enzymatic activity, suggesting that functional interdependence is mediated through domain-domain interactions rather than autonomous folding (Bai et al., 2019). Further investigations into *Pni*DAH7PS-CM demonstrate that catalysis involves dynamic interdomain motions within the homodimeric assembly, with polar interactions (e.g., hydrogen bonds and salt bridges) likely playing a dominant role in maintaining functional crosstalk (Bai and Parker, 2021). However, the absence of a high-resolution structural model has precluded precise characterization of heterodomain interaction networks in *Pni*DAH7PS-CM. These findings challenge the conventional view of enzyme modularity and raise questions about the biochemical basis of interdomain communication in chimeric enzymes.

Notably, *Porphyromonas gingivalis*, a keystone pathogen in periodontitis, harbors a homologous DAH7PS-CM enzyme (*Pgi*DAH7PS-CM) that shares high sequence identity with *Pni*DAH7PS-CM (Wu and Woodard, 2006). Preliminary studies confirm that splitting *Pgi*DAH7PS-CM into isolated DAH7PS and CM domains similarly results in near-complete loss of catalytic activity (Wu and Woodard, 2006). However, the structural determinants and functional mechanisms underlying this interdependency remain unresolved. Given the central role of *P. gingivalis* in periodontal disease pathogenesis and the absence of the shikimate pathway in humans, *Pgi*DAH7PS-CM represents a promising therapeutic target. Elucidating the molecular basis of its domain interdependence could enable strategies to disrupt bacterial aromatic amino acid biosynthesis, thereby attenuating both bacterial growth and virulence factor production, with potential applications in mitigating periodontal infections.

In this study, we investigate the structural and functional interdependency of the DAH7PS and CM domains in *Pgi*DAH7PS-CM. Using quaternary structure analysis of *Pgi*DAH7PS-CM combined with AlphaFold3’s high-quality predictions, we generated a structural model that delineates two distinct interdomain interfaces and their stabilizing interactions. Subsequent validation experiments, including analysis of catalytic activity and conformational changes under high Na^+^ concentrations (exploiting their kosmotropic effects and charge screening properties; Wiggins, 1996; Zhang and Cremer, 2006) and site-directed mutagenesis of key heterodomain interaction residues, confirmed the model’s reliability. These approaches further revealed the molecular basis of interdomain interactions and functional crosstalk in this bifunctional enzyme. Our findings not only provide mechanistic insights into the unique biochemistry of chimeric DAH7PS-CM enzymes but also highlight potential avenues for targeting these enzymes with salt-modulating inhibitors to combat periodontal pathogens.

This work bridges a critical knowledge gap in understanding the adaptive evolution of bifunctional enzymes in pathogenic bacteria and offers experimental evidence to guide the development of novel antimicrobial strategies against *P. gingivalis*-associated infections.

## 2 Materials and methods

### 2.1 Bioinformatics

The PgiDAH7PS-CM protein investigated in this study (UniProt ID: B2RJM7; Gene ID: PGN_RS05050) was classified into the DAH7PS-CM subfamily (PF00793-PF01817) based on domain architecture data retrieved from the Pfam database (Finn et al., 2013). Raw DAH7PS-CM protein sequences for multiple sequence alignment (MSA) were collected from Pfam and subjected to redundancy reduction using CD-HIT (Li et al., 2001), generating 697 non-redundant representative sequences. These sequences were aligned with Clustal Omega (Sievers et al., 2011), followed by targeted truncation to isolate CM domain sequences using Jalview (Waterhouse et al., 2009). Secondary structure predictions for the CM domain were generated via the JPred 4 server (Alexey et al., 2015).

### 2.2 Construction of expression vectors

The genes encoding *Pgi*DAH7PS-CM and its variant *Pgi*DAH7PS-CMVar were codon-optimized for expression in *E. coli* and commercially synthesized (Sangon Biotech, Shanghai, China). These synthetic constructs were amplified via PCR using sequence-specific primers (Supplementary Table 1), followed by restriction enzyme digestion and directional cloning into the pET28a expression vector through *Nde*I and *Xho*I restriction sites. This strategy enabled recombinant production of both enzymes featuring an N-terminal 6 × His tag followed by a TEV protease sequence to facilitate subsequent tag removal. Truncated variants *Pgi*DAH7PS and *Pgi*CM were generated through PCR-mediated domain isolation using *Pgi*DAH7PS-CM as template with designed primers (Supplementary Table 1). The amplified fragments were subcloned into the pET28a backbone via the same restriction sites for constructions of corresponding expression plasmids.

### 2.3 Protein expression and purification

The recombinant plasmids were introduced into *E. coli* BL21 (DE3) competent cells. Transformed cells were cultivated in lysogeny broth (LB) medium supplemented with 50 μg/mL kanamycin at 37°C with vigorous shaking until reaching mid-log phase (OD_600_ of 0.4 – 0.8). Protein expression was induced by adding 0.5 mM isopropyl β-D-1-thiogalactopyranoside (IPTG), followed by 16-h post-induction incubation at 23°C to facilitate proper folding. Harvested cells were pelleted via centrifugation (12,000 × g, 4°C, 15 min) and resuspended in ice-cold lysis buffer containing 50 mM Bistris propane (BTP, pH 7.4), 200 mM KCl, and 2 mM dithiothreitol (DTT). Cellular disruption was achieved through probe sonication (3 s pulse/7 s interval, 20 min duration) on ice, with subsequent clarification of lysates by centrifugation (40,000 × g, 4°C, 40 min).

The supernatant was filtered and loaded onto a pre-equilibrated HisTrap HP column (Cytiva) charged with Ni^2+^ ions, using 20 mM sodium phosphate buffer (pH 7.4) containing 250 mM NaCl and 20 mM imidazole as binding buffer. Target proteins were eluted with a linear imidazole gradient up to 500 mM in the same buffer system. Pooled eluates underwent buffer exchange using PD-10 desalting columns (Cytiva) to remove imidazole prior to tag cleavage. After overnight TEV protease digestion at 4°C, cleaved products were reapplied to the HisTrap column to separate the His-tagged TEV protease, free tags, and uncleaved proteins from the untagged target proteins.

### 2.4 Kinetic characteristics

Enzyme kinetic characterization of *Pgi*DAH7PS-CM and its variants was performed through optimized spectrophotometric quantification employing established methodologies (Schoner and Herrmann, 1976). Catalytic activities were monitored in real-time using a temperature-controlled UV-Vis spectrophotometer (37°C) with 1 cm pathlength quartz cuvettes, with DAH7PS activity quantified through PEP depletion at 232 nm and CM activity assessed via chorismate consumption at 274 nm. Reaction velocities were derived from linear absorbance changes and subsequently fitted to the Michaelis-Menten equation through nonlinear regression analysis to determine apparent kinetic parameters (*K*_*m*_ and *k*_*cat*_).

For DAH7PS kinetic profiling, reaction mixtures contained 50 mM BTP buffer (pH 7.4), 100 μM MnSO_4_ and 0.25 μM enzyme, with fixed substrate concentrations maintained at 750 μM for either PEP or E4P while systematically varying the complementary substrate concentration. CM activity assays were conducted under identical buffering conditions using 0.25 μM enzyme, initiated through chorismate addition across a concentration gradient spanning 10–300 μM. All reactions were performed in triplicate with appropriate blank corrections.

### 2.5 Determination of oligomeric state and molecular dimension

The oligomeric state and hydrodynamic properties of *Pgi*DAH7PS-CM and its variants were investigated through analytical size exclusion chromatography (SEC) coupled with calibrated molecular dimension analysis (Le Maire et al., 1986; Uversky, 1993). Chromatographic separations were performed using an ENrich SEC 650 column (Bio-Rad, CA) pre-equilibrated with 50 mM BTP buffer (pH 7.4) at 4°C. Molecular mass determination employed a six-component calibration set containing Vitamin B12 (1.35 kDa), myoglobin (17 kDa), ovalbumin (44 kDa), γ-globulin (158 kDa), thyroglobulin (670 kDa), and blue dextran (2,000 kDa), with each standard injected at 0.5 mg/mL. For Stokes radius (*R*_*s*_) calculations, an alternative calibration series was implemented using thyroglobulin (8.6 nm), aldolase (4.8 nm), ovalbumin (2.8 nm), myoglobin (1.9 nm), and cytochrome C (1.7 nm), following established methodologies.

Sample proteins were loaded at 1 mg/mL in SEC buffer and eluted isocratically at 0.2 mL/min, with elution volumes (V_*e*_) recorded relative to the void volume (V_0_) determined by blue dextran migration. The partition coefficient (*K*_*av*_) was calculated as (V_*e*_ - V_0_)/(V_*c*_ - V_0_), where Vc represents the total column volume. Molecular mass estimation involved plotting log-transformed standard masses (lgMW) against their corresponding *K*_*av*_ values to generate a linear regression model, enabling extrapolation of experimental protein masses from elution profiles. Experimental molecular masses were normalized against theoretical monomeric masses derived from sequence analysis to deduce oligomeric states. While hydrodynamic dimension analysis required transformation of the calibration strategy, with *K*_*av*_ values plotted against the logarithmic Stokes radii (lg*R*_*s*_) of the alternative standard series. This secondary calibration permitted calculation of experimental *R*_*s*_ values through inverse interpolation of sample retention characteristics.

### 2.6 Computational structural characterization and comparative analysis

The three-dimensional architecture of *Pgi*DAH7PS-CM was predicted *de novo* using AlphaFold3 (Abramson et al., 2024), employing its advanced deep learning framework for multi-chain protein structure modeling. Predicted models were visualized in PyMOL 4.6.0. Oligomeric interface characterization was performed through complementary computational approaches: PDBePISA (EMBL-EBI) for buried surface area quantification and interaction thermodynamics (Krissinel and Henrick, 2007), alongside PLIP (Adasme et al., 2021) for polar and hydrophobic contacts mapping. Comparative structural biology was conducted using the jFATCAT-flexible algorithm within the RCSB PDB Pairwise Alignment Tool (Zhanwen et al., 2020; Sebastian et al., 2024), aligning the predicted *Pgi*DAH7PS-CM structure against the crystallographically resolved the DAH7PS from *Pyrococcus furiosus* (*Pfu*DAH7PS, PDB ID: 4c1k) with iterative rigid-body and flexible loop adjustments. Structural similarity was quantified through root-mean-square deviation (RMSD) calculations for Cα atoms and template modeling score (TM-score) analysis.

## 3 Results

### 3.1 Domain architecture and dissection analysis of bifunctional *Pgi*DAH7PS-CM

Pfam analysis revealed that *Pgi*DAH7PS-CM comprises 366 amino acid residues, with the DAH7PS domain spanning residues 17 - 253 and the CM domain folded by residues 265 - 356. These two domains are connected by an 11-residue flexible linker (Supplementary Figure 1). To elucidate functional and structural heterodomain interdependencies, the DAH7PS and CM domains (*Pgi*DAH7PS and *Pgi*CM) were split between R258 and I259, and expressed and purified individually, followed by comparative kinetic and quaternary structural analyses.

Kinetic characterization demonstrated significant activity loss upon domain separation. For PgiDAH7PS, the *k*_*cat*_ decreased from 6.3 ± 0.6 s**^–^**^1^ in the full-length enzyme to 0.58 ± 0.05 s**^–^**^1^, while substrate affinities for DAH7PS substrates, PEP and E4P, were severely impaired, with *K*_*m*_ values increasing from 96 ± 8 μM to 515 ± 72 μM and 115 ± 10 μM to 718 ± 66 μM, respectively (Table 1). Similarly, the *K*_*m*_ for CM substrate, chorismite, rose from 19.1 ± 1.3 μM to 207 ± 25 μM in separated *Pgi*CM, though its *k*_*cat*_ remained largely unaffected (Table 1). These results indicate that domain separation disrupts catalytic efficiency for both enzymes, underscoring a profound functional interdependence between the DAH7PS and CM domains.

**TABLE 1:** Kinetic parameters for the DAH7PS and CM activities of *Pgi*DAH7PS-CM and its variants, and *Pgi*DAH7PS-CM in the absence or presence of 300 mM NaCl.

Enzyme	DAH7PS activity					CM activity		
	*K*_*m*_*^PEP^* (μM)	*K*_*m*_*^E4P^* (μM)	*k*_cat_ (s^–1^)	*k*_cat_/*K*_*m*_*^PEP^* (μM. s^–1^)	*k*_cat_/*K*_*m*_*^E4P^* (μM. s^–1^)	*K*_m_ (μM)	*k*_cat_ (s^–1^)	*k*_cat_/*K*_m_ (μM. s^–1^)
**Wild-type and truncated variants**						**Wild-type and truncated variants**		
***Pgi*DAH7PS*^WT^***	96 ± 8	115 ± 10	6.3 ± 0.6	6.6 × 10**^–^**^2^	5.5 × 10**^–^**^2^	19.1 ± 1.3	1.12 ± 0.03	5.9 × 10**^–^**^2^
***Pgi*DAH7PS*^D^***	515 ± 72	718 ± 66	0.58 ± 0.05	1.1 × 10**^–^**^3^	8.1 × 10**^–^**^4^	NA	NA	NA
***Pgi*DAH7PS*^CM^***	NA	NA	NA	NA	NA	207 ± 25	1.02 ± 0.10	4.9 × 10**^–^**^3^
**300 mM NaCl**						**300 mM NaCl**		
***Pgi*DAH7PS*^WT^***	NA	NA	NA	NA	NA	199 ± 15	1.04 ± 0.09	5.2 × 10**^–^**^3^
**E269A/E270A/E287A/R291A mutation**						**E269A/E270A/E287A/R291A mutation**		
***Pgi*DAH7PS*^Var^***	436 ± 46	511 ± 43	1.28 ± 0.11	2.9 × 10**^–^**^3^	2.5 × 10**^–^**^3^	81.1 ± 6.5	1.04 ± 0.08	1.3 × 10**^–^**^2^

The uncertainty values represent the standard deviation from triplicate measurements.

**FIGURE 2:**
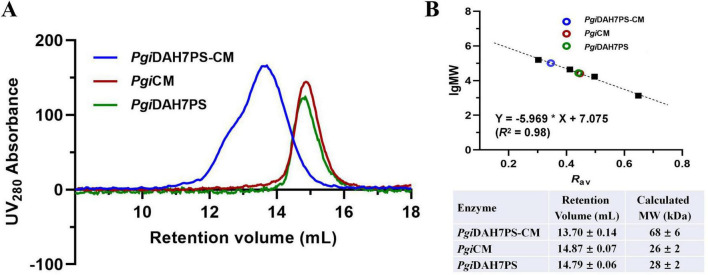
Molecular mass evaluation of *Pgi*DAH7PS-CM, *Pgi*DAH7PS and *Pgi*CM using analytical SEC. **(A)** SEC elution traces for ∼ 1 mg/mL *Pgi*DAH7PS-CM, *Pgi*DAH7PS, and *Pgi*CM. **(B)** Upper panel: Calibration plot, regression equation and goodness-of-fit evaluation based on SEC traces, with each point representing lgMW/*R*_av_ for standards (black solid squares) or samples (colored circles); Lower panel: Apparent molecular weights calculated from retention volumes. Errors represent standard deviations from three independent experiments.

Quaternary structural analysis via analytical SEC revealed distinct assembly patterns between *Pgi*DAH7PS-CM and its two truncated variants. The full-length *Pgi*DAH7PS-CM whose theoretical monomer MW is 42 kDa, was eluted at 13.70 ± 0.14 mL (Figure 2A), corresponding to a molecular weight (MW) of 68 ± 6 kDa (Figure 2B), consistent with a homodimeric assembly. In contrast, *Pgi*DAH7PS (theoretical monomer MW: 29 kDa) eluted at 14.79 ± 0.06 mL (Figure 2A), aligning with a monomeric state (28 ± 2 kDa) (Figure 2B), while *Pgi*CM (theoretical monomer MW: 13 kDa) eluted at 14.87 ± 0.07 mL (Figure 2A), matching a homodimer (26 ± 2 kDa) (Figure 2B). This suggests that the separated DAH7PS barrels lack homo-interactions intensive enough for oligomerization, whereas the CM domain adopts a canonical homodimeric quaternary structure. Therefore, the homodimeric assembly of full-length *Pgi*DAH7PS-CM appears to be predominantly maintained by the dimerization of CM domain. It should also be noted that the so-called disintegration of DAH7PS barrel homodimerization strictly refers to the dynamic equilibrium between monomer and dimer being dramatically shifted toward the monomer, thereby significantly decreasing the content of homodimeric species to levels undetectable by SEC under laboratory conditions.

### 3.2 *In silico* structural modeling and secondary structure validation of the *Pgi*DAH7PS-CM homodimes

**FIGURE 3:**
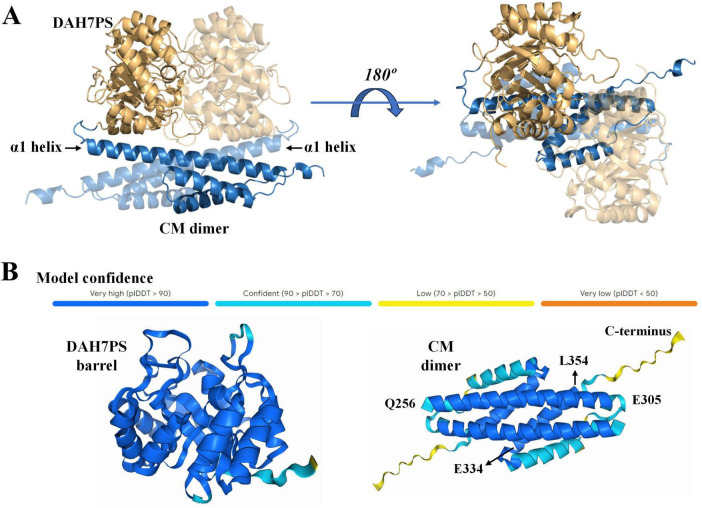
Predication of 3D structure of *Pgi*DAH7PS **(A)** and structural confidence evaluation **(B)** by AlphaFold3. In **(A)**, DAH7PS and CM domains are respectively showed by blue and yellow cartoons. Two monomeric units are differentiated by varied transparency.

To elucidate the molecular mechanism underlying the functional interplay between the DAH7PS and CM domains, we generated a structural model for the *Pgi*DAH7PS-CM homodimer using AlphaFold3 open sever (Figure 3A). In this model, the CM domains from both protomers adopt a triple-helix bundle fold and assemble into a homodimeric configuration, as anticipated (Figure 3A). In contrast, the DAH7PS domains exhibit the canonical TIM barrel architecture, with two barrels forming a dimer-like assembly positioned adjacent to the α1 helices of the CM dimer. The overall architecture of the *Pgi*DAH7PS-CM homodimer displays a centrosymmetric arrangement (Figure 3A).

The AlphaFold3 prediction yielded high confidence scores for the majority of the model, with the exception of the helix α3 and the unstructured C-terminal region in the CM domain, which showed lower confidence values of 70–90% and 50–70%, respectively (Figure 3B). Secondary structure analysis of *Pgi*CM via JPred corroborated AlphaFold3’s prediction of a triple-helix bundle topology in the CM domain. Residue segments Q256–E305 and E334–L354 were reliably predicted to adopt helices α1 and α3 (confidence scores > 0.9), while the C-terminal 15 residues (∼ 330 to 345) displayed no stable secondary structural elements (Figure 3B and Supplementary Figure S2). Based on this concordance between computational predictions, we adopted the AlphaFold3 model as a reliable framework for probing intramolecular interactions within the *Pgi*DAH7PS-CM homodimer.

### 3.3 Structural characterization of interdomain interfaces in *Pgi*DAH7PS-CM

**FIGURE 4:**
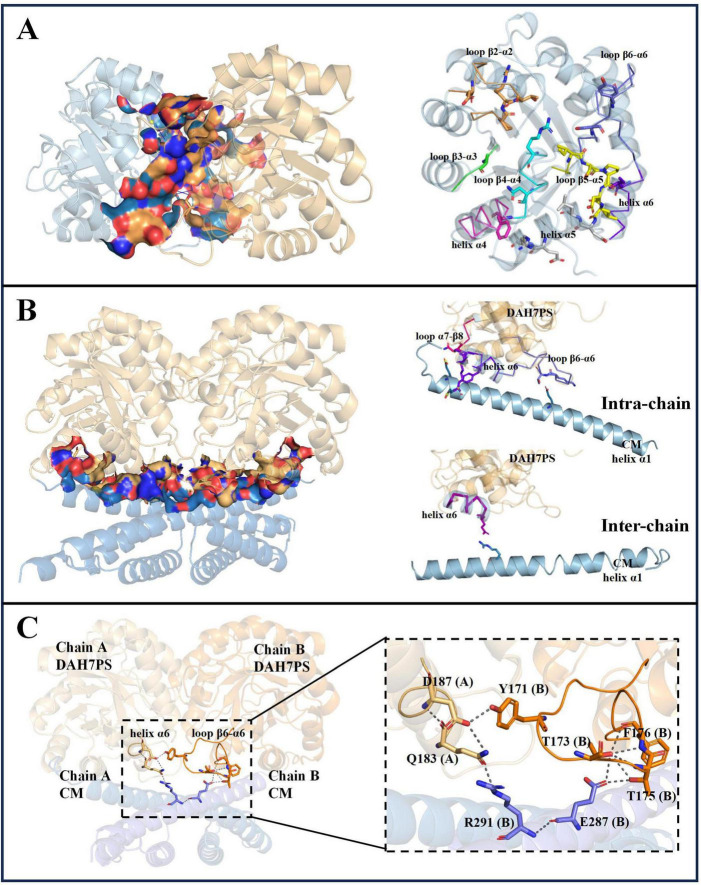
Analysis of interdomain interface in *Pgi*DAH7PS-CM homodimer. **(A)** Interface between two DAH7PS domains (left), and secondary structural elements (highlighted in colored ribbon) with residues forming the D-D interface (right). **(B)** Hetero-interface between DAH7PS and CM domains (left), and secondary structural elements (color-coded ribbons) containing residues positioned at D-CM intra-chain/inter-chain sub-interface (right). **(C)** Polar interaction networks linking key structural elements of DAH7PS and CM active sites in the homodimer model.

The structural model of *Pgi*DAH7PS-CM revealed two functionally distinct interaction interfaces critical for its architecture and catalytic regulation. The first interface emerges between two DAH7PS barrels (D-D interface), exhibiting an extensive interaction area of ∼ 1451 Å^2^ with a calculated free energy gain at complexation (ΔG*^s^*) of –18.6 kcal/mol (Figure 4A). This interface is stabilized through complementary polar and hydrophobic interactions involving conserved structural motifs: loop β2-α2 (P55, R56, T57), loop β4-α4 (R110, N114, F116), loop β5-α5 (I139 - L143), helix α5 (D144, L145, E151, R152), and loop β6-α6 (S169, Y171) (Figure 4A and Table 2). Notably, loops β2-α2, β4-α4, and β6-α6 do not only contribute to the DAH7PS homo-interaction but also form essential components of the DAH7PS’s active site responsible for substrate binding (PEP and E4P). Particularly, the direct participation of E4P-binding residues (P55, R56, T57) in interface formation further suggests a structural coupling between dimer stabilization and active site conformation (Figure 4A; Table 2; Supplementary Figure 2). This spatial overlap implies that D-D interactions may dynamically modulate the catalytic microenvironment.

**TABLE 2:** Hydrogen bonds and salt bridges maintaining D-D and D-CM interfaces in the *Pgi*DAH7PS-CM model.

D-D interface				D-CM interface			
H-bond residue pairs		Salt-bridge residue pairs		H-bond residue pairs		Salt-bridge residue pairs	
Arg56_A_	Arg152_B_	Glu63_A_	Arg152_B_	**Intra-chain**		**Intra-chain**	
Glu6_A_	Arg152_B_	Glu63_A_	Arg152_B_	Thr173_A_	Glu287_A_	Lys189_A_	Glu269_A_
Glu63_A_	Arg152_B_	Asp142_A_	Arg110_B_	Thr175_A_	Glu287_A_	Arg190_A_	Glu270_A_
Ser140_A_	Arg110_B_	Asp142_A_	Arg110_B_	Asn222_A_	Glu269_A_	Lys189_B_	Glu269_B_
Ser140_A_	Ser169_B_	Arg152_A_	Glu63_B_	Thr173_B_	Glu287_B_	Arg190_B_	Glu270_B_
Asp142_A_	Arg110_B_	Arg152_A_	Glu63_B_	Thr175_B_	Glu287_B_		
Asp187_A_	Tyr171_B_	Arg110_A_	Asp142_B_	Asn222_B_	Glu269_B_		
Arg152_A_	Arg56_B_	Arg110_A_	Asp142_B_	**Inter-chain**		**Inter-chain**	
Arg152 _A_	Glu63_B_			Gln183_A_	Arg291_B_		
Arg15_A_	Glu63_B_			Arg291_A_	Gln183_B_		
Arg110_A_	Ser140_B_						
Arg110_A_	Asp142_B_						
Ser169_A_	Ser140_B_						
Tyr171_A_	Asp187_B_						

Subscripts denote protein chain numbers, while white and grey backgrounds highlight residues corresponding to the DAH7PS and CM domains, respectively.

A second interface bridges the DAH7PS barrels and the CM dimer (D-CM interface), spanning around 1,560 Å^2^ with a ΔG*^s^* of –16.6 kcal/mol (Figure 4B). Polar interactions dominate this interface, organized into two distinct sub-regions (Table 2). The intra-chain sub-interface involves DAH7PS structural elements (loop β6-α6: T173, T175; helix α6: K189, R190; loop α7-β8: N222) and the CM domain’s helix α1 (E269, E270 and E287) within the same protomer (Figure 4B and Table 2). In contrast, the inter-protomer sub-interface engages DAH7PS helix α6 (Q183) and CM helix α1 (R291) from the adjacent protomer (Figure 4B; Table 2). Strikingly, loop β6-α6, a critical active site element of DAH7PS as aforementioned, serves multiple roles by contributing to substrate coordination and both D-D and D-CM interface formations (Figures 4A,B). Structural alignment further identified that the residue R291 electrostatically coupled with the end of loop β6-α6 (Q183) is positioned adjacent to the conserved active-site residue R292 of the CM domain, suggesting a direct functional crosstalk between the two enzymatic domains potentially (Figure 4C and Supplementary Figure 3).

### 3.4 Validation of AlphaFold-predicted domain interfaces through NaCl-modulated catalytic activity and conformational expansion

Structural analysis employing AlphaFold-predicted models demonstrated that polar contacts predominantly stabilize both the D-D and D-CM interfaces of *Pgi*DAH7PS-CM, indicating their critical role in maintaining catalytic competence. Building on prior studies demonstrating that Na^+^ ions act as kosmotropic agents to disrupt protein-protein hydrogen bonds through water competition and attenuate electrostatic interactions via charge screening at elevated concentrations (Wiggins, 1996; Zhang and Cremer, 2006), we systematically evaluated the structural-functional dependence on polar contacts by quantifying catalytic parameters and monitoring conformational alteration under increased NaCl concentrations.

In the absence of NaCl, PgiDAH7PS-CM exhibited robust DAH7PS activity, whereas this activity was nearly abolished under 300 mM NaCl. For the CM domain, detectable activity persisted but was severely attenuated. Kinetic analysis revealed a 10-fold increase in the *K*_*m*_ value for chorismate (from 19.1 ± 1.3 μM to 199 ± 15 μM) and a marginal reduction in *k*_*cat*_ (from 1.12 ± 0.03 to 1.04 ± 0.09 s^–1^), indicating impaired substrate binding without significant alteration of catalytic turnover (Table 1).

**FIGURE 5:**
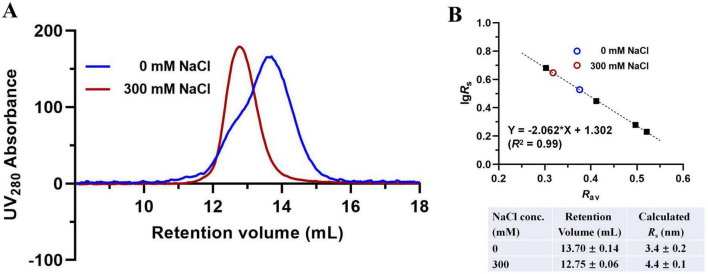
Molecular dimension evaluation of *Pgi*DAH7PS-CM in the absence or presence of 300 mM NaCl. **(A)** SEC elution traces for ∼ 1 mg/mL *Pgi*DAH7PS-CM in 0 or 300 mM NaCl. **(B)** Upper panel: Calibration plot, regression equation and goodness-of-fit evaluation based on SEC traces, with each point representing lg*Rs*/Rav for standards (black solid squares) or samples (colored circles); Lower panel: Calculated *R*_s_ values corresponding to retention volumes. Column equilibration and calibration with standards were repeated in the absence or presence of 300 mM NaCl, which showed insignificant influence of NaCl concentration on retention volumes of the standards. Errors represent standard deviations from three independent experiments.

The AlphaFold model predicts that disruption of the D-D and D-CM interfaces would destabilize the two DAH7PS barrels, leading to their dissociation from the CM dimer. This structural separation is hypothesized to increase the overall molecular dimensions of the protein. Supporting this notion, prior studies on PniDAH7PS-CM revealed significant conformational polymorphism, with radius of gyration (*R*_*g*_) values ranging from 30 to 46 Å, reflecting its structural flexibility (Bai et al., 2019; Bai and Parker, 2021). To probe the conformational changes in *Pgi*DAH7PS-CM under high ionic strength, we performed analytical SEC in the presence of 300 mM NaCl. Retention volumes were converted to Stokes radius, *R*_*s*_, defined as the radius of a hypothetical sphere with equivalent hydrodynamic properties to the biomolecule (Le Maire et al., 1986; Uversky, 1993). Under NaCl-free conditions, *Pgi*DAH7PS-CM exhibited an *R*_*s*_ value of 3.4 ± 0.2 nm, consistent with a compact conformation stabilized by extensive interdomain interactions (Figure 5). In contrast, at 300 mM NaCl, the *R*_*s*_ increased to 4.4 ± 0.1 nm, indicating a markedly extended conformation (Figure 5). This expansion aligns with the predicted dissociation of DAH7PS barrels from the CM dimer and suggests that high Na^+^ concentrations destabilize domain interfaces, disfavoring the DAH7PS-CM assembly required for optimal catalytic function.

### 3.5 Mutagenesis analysis of CM domain polar contact residues and their impact on *Pgi*DAH7PS-CM catalytic activities and structural stability

Structural analysis identified four polar contact-forming residues (E269, E270, E287, R291) in helix α1 of CM domain that interact with DAH7PS barrels at the D-CM interface. To investigate their functional roles, the four residues were all mutated to Ala (E269A/E270A/E287A/R291A), generating variant *Pgi*DAH7PS-CM*^Var^*.

Enzyme kinetics demonstrated substantial impairment of CM activity in *Pgi*DAH7PS-CM*^Var^*, with chorismate binding affinity decreasing 4.2-fold (*K*_*m*_ = 81.1 ± 6.5 μM vs wild-type 19.1 ± 1.3 μM), accompanied by a modest 7% reduction in turnover number, from a *k*_*cat*_ value of 1.12 ± 0.03 s^–1^ to 1.04 ± 0.08 s^–1^ (Table 1). Strikingly, DAH7PS activity was concurrently compromised, showing ∼ 4.5- and 4-fold increases in apparent *K*_*m*_ values for PEP (436 ± 46 μM) and E4P (511 ± 43 μM), respectively, along with reduced *k*_*cat*_ from 6.3 ± 0.6 s^–1^ to 1.28 ± 0.11 s^–1^ (Table 1).

**FIGURE 6:**
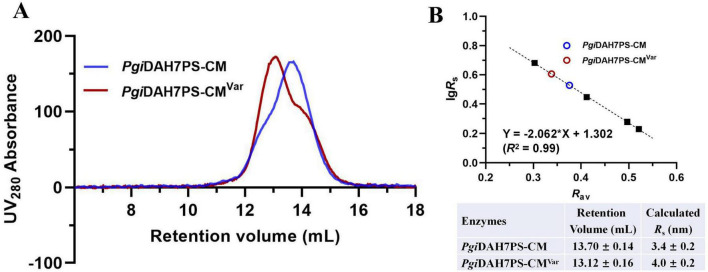
Molecular dimension evaluation of *Pgi*DAH7PS-CM and *Pgi*DAH7PS-CM*^Var^*. **(A)** SEC elution traces for ∼ 1 mg/mL *Pgi*DAH7PS-CM and *Pgi*DAH7PS-CM*^Var^*. **(B)** Upper panel: Calibration plot, regression equation and goodness-of-fit evaluation based on SEC traces, with each point representing lg*Rs*/Rav for standards (black solid squares) or samples (colored circles); Lower panel: Calculated *R*_s_ values corresponding to retention volumes. Errors represent standard deviations from three independent experiments.

SEC analysis revealed pronounced structural perturbations, with *Pgi*DAH7PS-CM*^Var^* exhibiting decreased retention volume of 13.12 ± 0.16 mL compared to the 13.70 ± 0.14 mL for wild-type enzyme (Figure 6). This change in retention volumes was correspondent to an increased *R*_*s*_ from 3.4 ± 0.2 to 4.0 ± 0.2 nm due to the quadruple mutations (Figure 6B), being indicative of a transition from a compact to an extended conformation of *Pgi*DAH7PS-CM.

Collectively, the observed disruption in both CM and DAH7PS catalytic functions, coupled with altered structural compactness of *Pgi*DAH7PS-CM, validated that residues E269, E270, E287, and R291, through their interdomain polar contacts, are critical for preserving the structural integration and heterodomain functional interdependence of *Pgi*DAH7PS-CM.

## 4 Discussion

The DAH7PS-CM enzyme subclass, exemplified by *Pgi*DAH7PS-CM and *Pni*DAH7PS-CM, appears predominantly in periodontal pathogens such as *Porphyromonas* and *Prevotella* species (Finn et al., 2013; Bai et al., 2019). This chimeric enzyme exhibits unique architectural features distinguishing it from conventional bifunctional enzymes. While typical bifunctional enzymes catalyze sequential reactions through spatially adjacent catalytic centers often connected by substrate channels (Anderson et al., 1991; Hu et al., 1992; Eberhard et al., 1995), DAH7PS-CM mediates non-consecutive catalytic steps in the shikimate pathway. Notably, although CM-DAH7PS fusion proteins with N-terminal CM domains exist in other bacterial species (e.g., *Bacillus subtilis* and *Listeria monocytogenes*), these configurations primarily utilize the CM domain for allosteric regulation rather than direct catalytic cooperation (Light et al., 2012; Nazmi et al., 2016). The observed functional codependence between DAH7PS and CM moieties in DAH7PS-CM enzymes suggests evolutionary selection for this specific gene fusion to achieve coordinated catalytic regulation.

**FIGURE 7:**
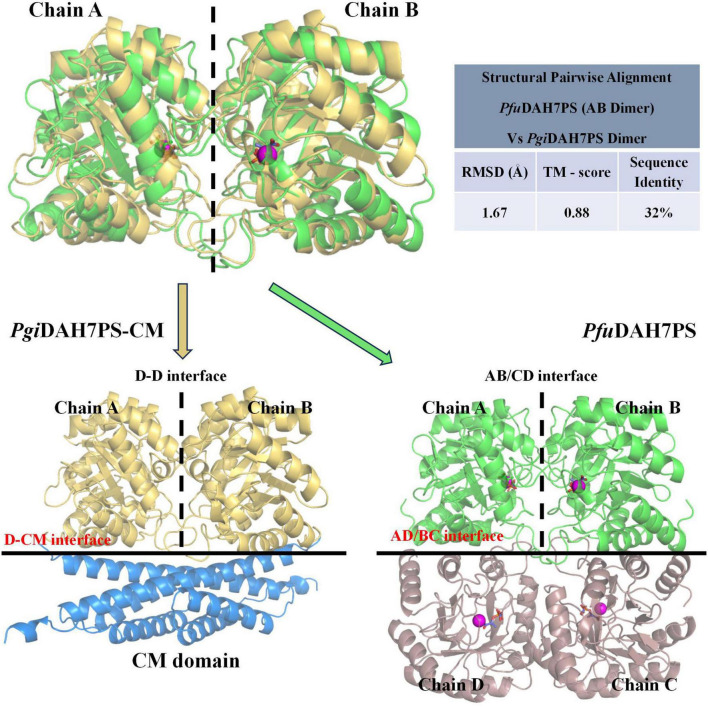
Structural comparison between interfaces in *Pgi*DAH7PS-CM and *Pfu*DAH7PS (PDB ID: 4c1k). Top panel: Pairwise structural alignment of *Pgi*DAH7PS dimer (chains A/B) and *Pfu*DAH7PS dimer (chains A/B). Alignment quality was assessed using: RMSD: Calculated from superimposed Cα backbones, with lower values indicating better geometric overlap. TM-score: Measures topological similarity (range: 0–1), where higher values reflect stronger structural conservation. Bottom panel: Interface comparison between the D-CM interface of *Pgi*DAH7PS-CM (left) and the AD/BC interface of *Pfu*DAH7PS (right). In *Pfu*DAH7PS, magenta spheres represent Cd^2+^ ions, and orange sticks depict PEP substrates.

Previous studies established that DAH7PS enzymes universally function as homodimers or homotetramers, with no reported hetero-oligomeric activation mechanisms (Jensen et al., 2002). As a representative of the well-studied DAH7PS enzymes, *Pfu*DAH7PS is assembled as a homotetramer. This structure exhibits two distinct interfaces: The AB/CD interface (chains A-B/C-D), proximal to the active site and enriched with catalytically essential residues (Figure 7; Nazmi et al., 2014), and the AD/BC interface (chains A-D/B-C) characterized by robust hydrophobic interactions distal from the catalytic center (Figure 7; Nazmi et al., 2014). Notably, this BC interface demonstrates substantial thermodynamic stability with a solvation energy, ΔG*^s^* of –35.4 kcal/mol, suggesting its critical role in stabilizing the adjacent AB interface through long-range structural reinforcement.

Building on this framework, structural alignment reveals remarkable evolutionary conservation in *Pgi*DAH7PS-CM. The D-D interface shares significant homology with *Pfu*DAH7PS’s AB interface, maintaining analogous roles in active site organization (Figure 7). Intriguingly, the *Pgi*CM dimer appears to functionally substitute for the B/D dimer observed in *Pfu*DAH7PS (Figure 7), though with distinct interaction characteristics. While the D-CM interface exhibits weaker interaction metrics (smaller contact area, ΔG*^s^* = –16.6 kcal/mol kJ/mol) dominated by polar contacts (Figure 4B and Table 2), its importance is underscored by DAH7PS domain dimer dissociation and activity loss upon domain separation (Figure 2). Exposure to elevated NaCl concentrations and mutagenesis of key CM residues (E269A/E270A/E287A/R291A) that mediate polar contacts with the DAH7PS domain both induce functional attenuation and conformational alterations in *Pgi*DAH7PS (Figures 5, 6; Table 1). These experimental perturbations collectively demonstrate the indispensable role of the D-CM interface in preserving the oligomeric state and catalytic capacity of the DAH7PS domain. Furthermore, the NaCl-mediated functional impairment reveals that polar contacts likely contribute critically to both D-D interface stability and substrate binding competence (Figure 4A; Table 2). Additionally, it is noteworthy that the SEC elution trace of *Pgi*DAH7PS-CM displays a shoulder at 12.5–13 mL elution volume. Having excluded interference from high-molecular-weight contaminants through SDS-PAGE analysis (Supplementary Figure 4), we postulate that wild-type *Pgi*DAH7PS-CM under these experimental conditions maintains an observable equilibrium between association and dissociation of DAH7PS-CM interdomain interactions. The minor dissociated population of *Pgi*DAH7PS-CM exhibits a smaller elution volume, analogous to the elution profiles observed for both *Pgi*DAH7PS-CM under high NaCl interference and *Pgi*DAH7PS-CMVar (Figures 5A, 6A).

The interfacial network extends beyond structural stabilization to direct catalytic modulation. At the D-CM interface, key DAH7PS active site elements, including the catalytically essential β6-α6 loop and α6 helix (T173, T175, Q183), form hydrogen bonds and salt bridges with CM residues E287 and R291 (Figure 4C). This intricate connectivity suggests conformational coupling between domain interaction and active site geometry of DAH7PS domain. In contrast, the D-CM interface does not play a decisive role in CM domain dimerization though (Figure 2), reciprocal effects arising from the D-CM hetero-interaction manifest in the CM domain. Specifically, interfacial residues E269, E270, and R291 are positioned near the catalytic center (Supplementary Figure 3). The strategic localization of R291, directly adjacent to the conserved catalytic residue R290, implies that the D-CM interface mediates conformational adjustments of the CM active site. Both domain separation and mutations (E269A/E270A/E287A/R291A) disrupt CM catalytic activity, unequivocally demonstrating the critical functional role of the D-CM interface (Figure 6; Table 1).

In summary, this interdependence manifests as an integrated stabilization mechanism where the D-D interface preserves DAH7PS active site architecture through evolutionarily conserved interactions, while the D-CM interface maintains quaternary structure through polar networks that simultaneously enable interdomain communication. Shared structural elements (β6-α6 loop) and interfacial residues (E287/R291) create a contiguous interaction network bridging the catalytic centers (Figure 4C), spatially coordinating DAH7PS’s pathway-initiating function with CM’s downstream activity. This model aligns with observed catalytic cooperativity in *Pni*DAH7PS-CM, where DAH7PS catalysis enhances CM catalytic efficiency in companion with global conformational changes (Bai and Parker, 2021), suggesting synchronized catalytic cycles mediated by allosteric crosstalk.

The phylogenetic specificity of DAH7PS-CM enzymes to periodontopathic bacteria acquires particular significance given the shikimate pathway’s essential role in bacterial survival and virulence factor production. The enzyme’s unique interfacial architecture, combining structural stabilization with functional coordination, presents dual targeting opportunities, either catalytic domain or their critical interaction networks, for developing novel therapeutics against *Porphyromonas* and *Prevotella*-associated periodontal diseases.

## Data Availability

The original contributions presented in the study are included in the article/Supplementary material, further inquiries can be directed to the corresponding authors.
