# Supplementary material for: Bidirectional interdomain crosstalk in a Porphyromonas gingivalis chimeric enzyme coordinates catalytic synergy for aromatic amino acid biosynthesis
Source: Front Microbiol. 2025 Jun 13;16:1601098. doi: 10.3389/fmicb.2025.1601098 (PMC12202928; doi:10.3389/fmicb.2025.1601098)
Supplement: Supplementary file 1 [file Supplementary_file_1.docx]

Supplementary Material

# Supplementary Tables

**Supplementary Table 1.** Primers for construction of expression vectors

| **Generic Primer for the addition of TEV site at 5’ end**  5’ AAACATATGGAAAACCTGTATTTTCAGGGCAGCGG 3’ |
| --- |
| **Primers for *Pgi*DAH7PS-CM and *Pgi*DAH7PS-CM^Var^**  Forward primer：5’ TTTTCAGGGCAGCGGCGCGATGAAGTACTGTGATTTTAC 3’  Reverse primer：5’ AAACTCGAGTCATGATATACAGTCGTCGGTGTGTG 3’ |
| **Primers for *Pgi*DAH7PS**  Forward primer：5’ TTTTCAGGGCAGCGGCGCGATGAAGTACTGTGATTTTAC 3’  Reverse primer：5’ AAACTCGAGTCATGAACGCAGTCGCCGGAGG 3’ |
| **Primers for *Pgi*CM**  Forward primer：5’ TTTTCAGGGCAGCGGCGCGATCCCACGCCGCCAATCCG 3’  Reverse primer：5’ AAACTCGAGTCATGATATACAGTCGTCGGTGTGTG 3’ |

CATATG: *Nde I* restriction site; CTCGAG: *Xho I* restriction site; GAAAACCTGTATTTTCAGGGC: TEV site.

**Supplementary Table 2.** Key residues mediating hydrophobic interactions at D-D and D-CM interfaces in the *Pgi*DAH7PS-CM structural model.

| **Residues involved in hydrophobic interaction** | |
| --- | --- |
| **D-D Interface** | **D-CM Interface** |
| Pro55 | Phe186 |
| Thr57 | Glu269 |
| Pro59 |  |
| Ala89 |  |
| Phe116 |  |
| Ile139 |  |
| Pro141 |  |
| Leu143 |  |
| Asp144 |  |
| Leu145 |  |
| Tyr171 |  |
| Ile184 |  |

White and grey backgrounds highlight residues corresponding to the DAH7PS and CM domains, respectively.

# Supplementary Figures


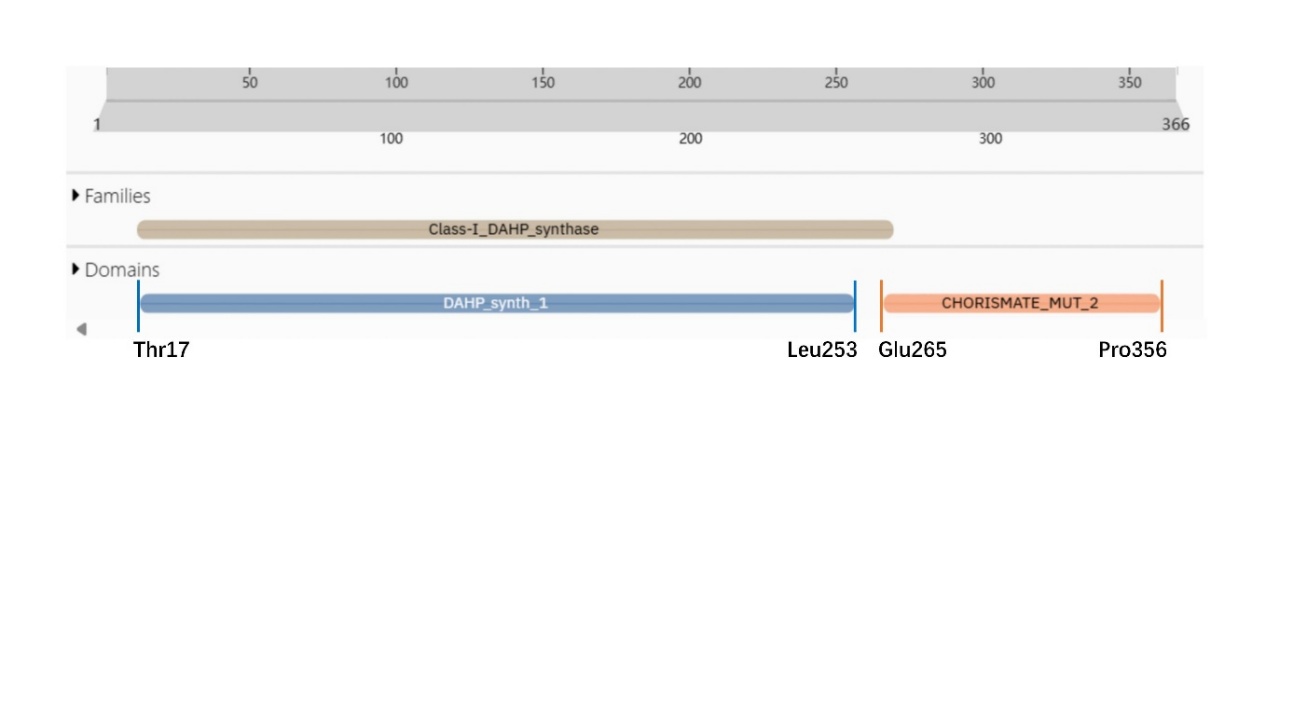


**Supplementary Figure 1.** Domain architecture of *Pgi*DAH7PS-CM predicted by Pfam.


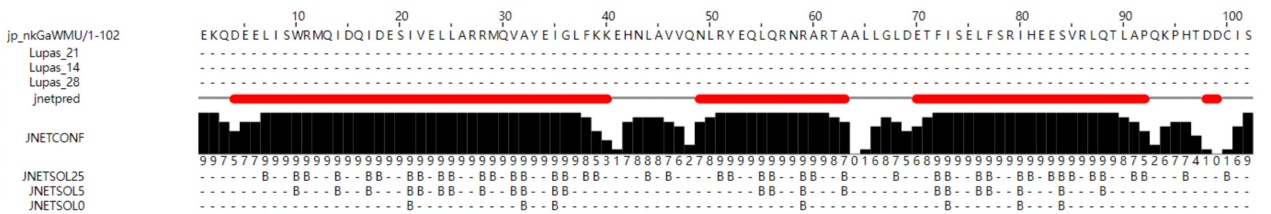


**Supplementary Figure 2.** Secondary structure of *Pni*CM predicted by JPred 4.


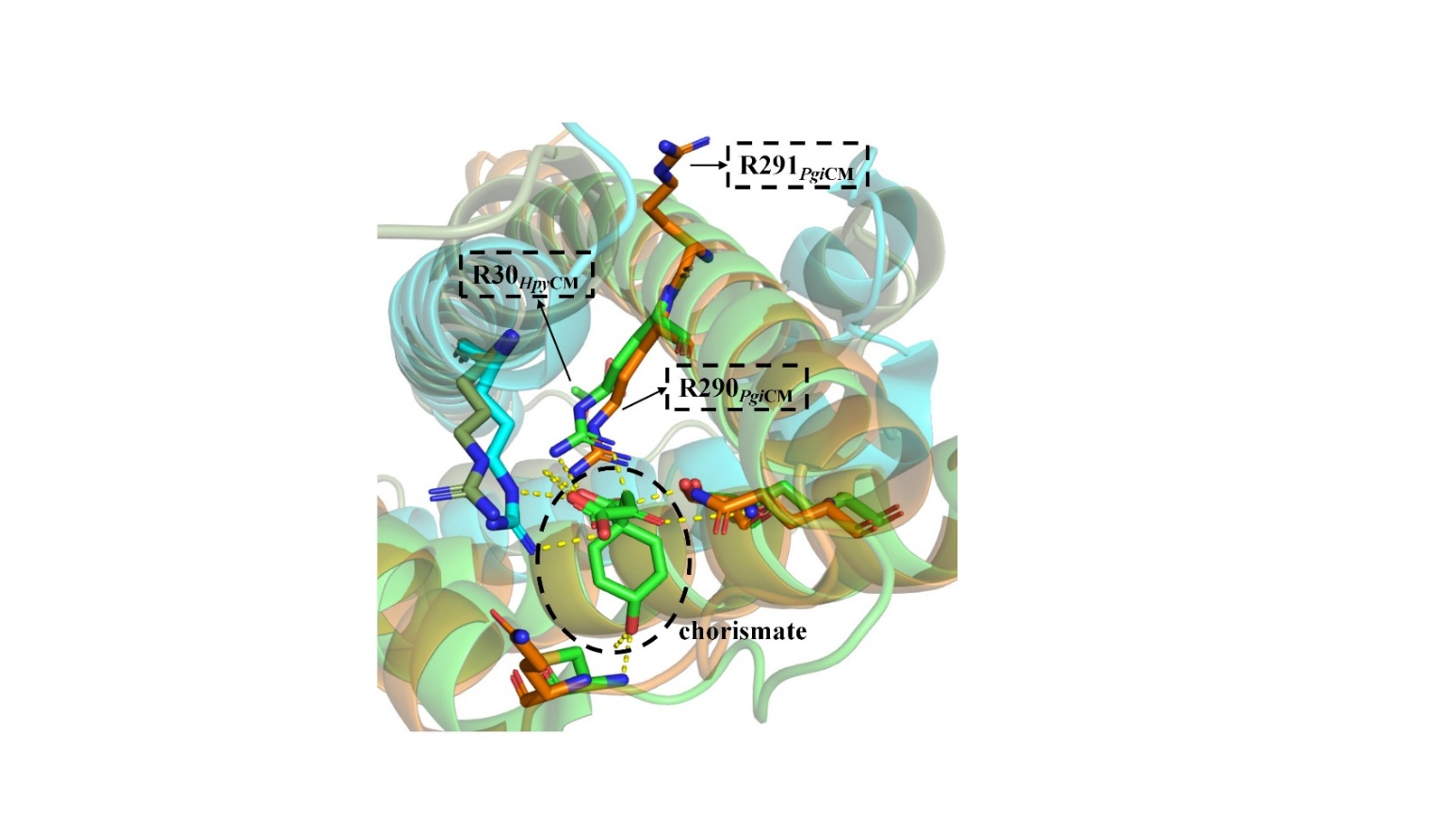


**Supplementary Figure 3.** Structural comparison between *Pgi*CM and the CM from *Helicobacter pylori* (*Hpy*CM, PDB ID: 6al9), highlighting the predicted active site architecture of *Pgi*CM. Subunits of *Pgi*CM are colored smudge and orange, while *Hpy*CM subunits are cyan and green. Functionally conserved residues (*Pgi*CM R290 ↔ *Hpy*CM R30) and D-CM interface residue R291 in *Pgi*CM are boxed with dashed lines. Prephenate, the product of the CM-catalyzed reaction, is shown bound to *Hpy*CM within dashed ellipse.


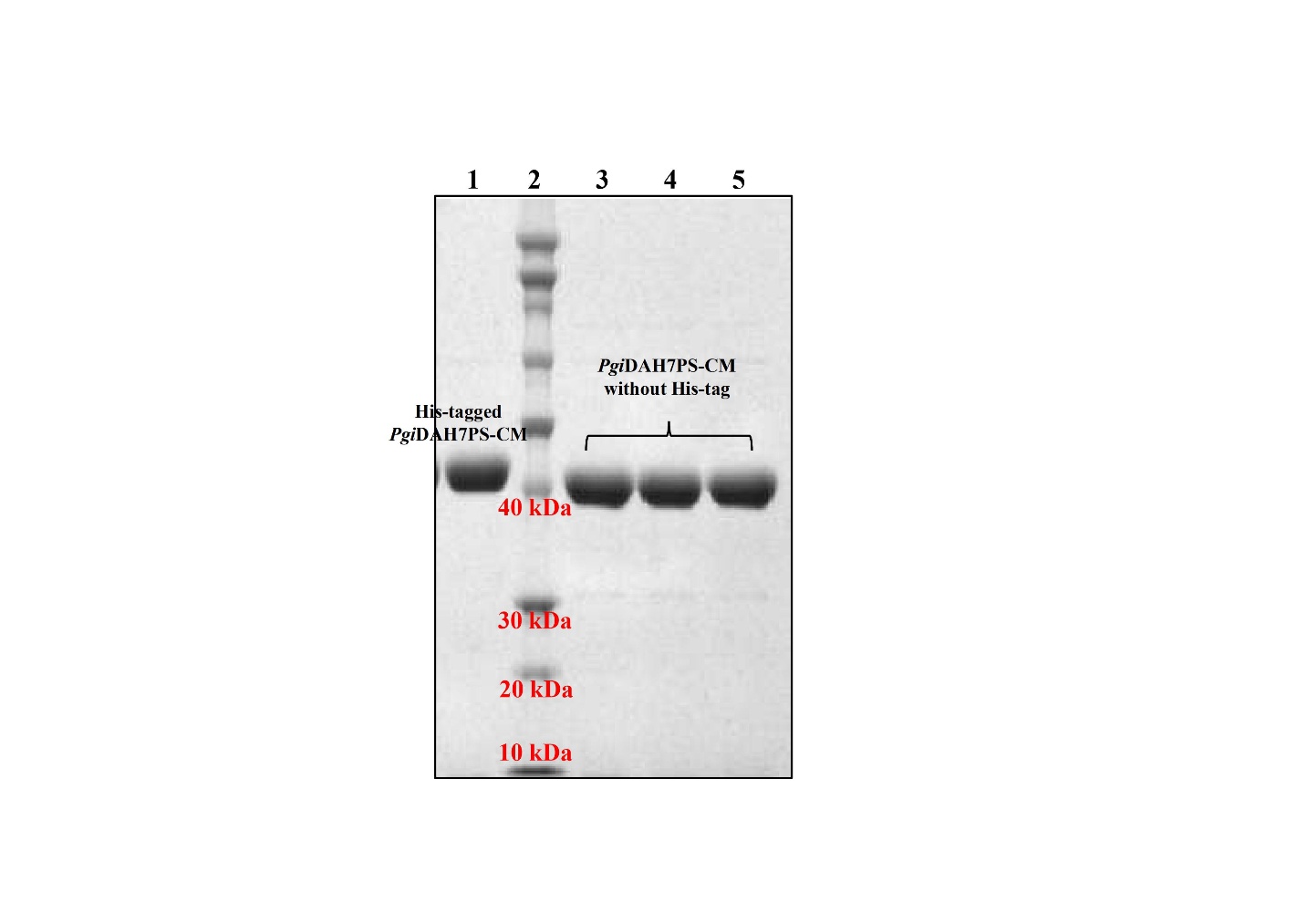


**Supplementary Figure 4.** The SDS-PAGE gel demonstrates the purity of purified *Pgi*DAH7PS-CM. Lane 1: HisTrap-purified *Pgi*DAH7PS-CM with His-tag; Lane 2: Protein standard marker; Lanes 3-5: Flow-through fractions from HisTrap after TEV protease digestion, showing His-tag-free pure *Pgi*DAH7PS-CM protein.

# Sequences

***Pgi*DAH7PS-CM (Protein sequence):**

MKYCDFTPLPLPSEPNTTVIAGPCSAESEEQIMTTARALRDEAGIRIFRAGLWKPRTLPGCFEGVGETGLPWLVRVQDELDMLATTEVATREHVEQAMQAGIRILWLGARTTSNPFAVQEIADTIGKDESVIVLVKNPISPDLDLWTGALERLRQSGVRQIGAIHRGFSTYATKTFRNPPHWQIPFDLKRRFPSLTILCDPSHITGQRDRIESVSQQAMEMNFDGLIIESHCCPDKALSDASQQITPTVLAQILRRLRIPRRQSEKQDEELISWRMQIDQIDESIVELLARRMQVAYEIGLFKKEHNLAVVQNLRYEQLQRNRARTAALLGLDETFISELFSRIHEESVRLQTLAPQKPHTDDCIS

***Pgi*DAH7PS-CM (DNA sequence):**

ATGAAGTACTGTGATTTTACGCCGTTGCCTCTCCCCTCGGAGCCTAATACGACAGTCATTGCCGGCCCTTGCAGTGCAGAAAGTGAGGAGCAGATAATGACTACTGCTCGTGCCCTCAGGGATGAAGCAGGCATTCGTATTTTTCGTGCCGGTCTGTGGAAACCTCGTACCTTGCCGGGGTGCTTCGAAGGAGTAGGAGAAACAGGGCTACCTTGGTTGGTGCGTGTACAGGATGAATTGGATATGCTTGCTACCACGGAAGTGGCTACTCGCGAACACGTAGAGCAAGCCATGCAAGCCGGTATCAGAATACTCTGGTTAGGTGCACGAACCACATCCAATCCCTTTGCTGTACAAGAAATTGCCGATACGATAGGCAAGGACGAATCGGTGATTGTCCTCGTCAAGAATCCGATCAGTCCCGATTTGGATCTGTGGACAGGAGCCCTAGAACGGCTTCGGCAGTCCGGAGTTCGACAGATCGGAGCCATCCATAGAGGATTCAGTACCTATGCGACCAAGACGTTTCGCAATCCTCCACATTGGCAGATTCCCTTCGATTTGAAAAGACGTTTTCCTTCGTTAACCATCCTTTGTGATCCGAGTCATATTACGGGACAGAGAGATCGGATCGAATCCGTCAGCCAGCAAGCCATGGAAATGAATTTTGACGGGCTGATCATTGAGTCGCATTGCTGTCCGGATAAGGCTCTAAGCGATGCAAGCCAGCAGATAACACCTACTGTACTTGCCCAAATCCTCCGGCGACTGCGTATCCCACGCCGCCAATCCGAAAAGCAGGACGAAGAGCTGATCTCTTGGCGCATGCAGATTGATCAGATAGATGAGAGTATAGTGGAATTGCTAGCTCGGCGGATGCAAGTGGCATACGAGATAGGTTTGTTCAAAAAAGAGCACAATCTGGCTGTGGTTCAGAATCTCCGCTACGAACAACTACAGCGCAACCGTGCCCGTACTGCAGCCCTCTTAGGTTTGGACGAAACATTTATATCGGAGCTATTCAGCCGTATTCATGAGGAATCTGTCCGTCTGCAGACCCTTGCCCCCCAAAAGCCACACACCGACGACTGTATATCATGA

***Pgi*DAH7PS-CM^Var^ (Protein sequence):**

MKYCDFTPLPLPSEPNTTVIAGPCSAESEEQIMTTARALRDEAGIRIFRAGLWKPRTLPGCFEGVGETGLPWLVRVQDELDMLATTEVATREHVEQAMQAGIRILWLGARTTSNPFAVQEIADTIGKDESVIVLVKNPISPDLDLWTGALERLRQSGVRQIGAIHRGFSTYATKTFRNPPHWQIPFDLKRRFPSLTILCDPSHITGQRDRIESVSQQAMEMNFDGLIIESHCCPDKALSDASQQITPTVLAQILRRLRIPRRQSEKQDAALISWRMQIDQIDESIVALLAARMQVAYEIGLFKKEHNLAVVQNLRYEQLQRNRARTAALLGLDETFISELFSRIHEESVRLQTLAPQKPHTDDCIS

***Pgi*DAH7PS-CM^Var^ (DNA sequence):**

ATGAAGTACTGTGATTTTACGCCGTTGCCTCTCCCCTCGGAGCCTAATACGACAGTCATTGCCGGCCCTTGCAGTGCAGAAAGTGAGGAGCAGATAATGACTACTGCTCGTGCCCTCAGGGATGAAGCAGGCATTCGTATTTTTCGTGCCGGTCTGTGGAAACCTCGTACCTTGCCGGGGTGCTTCGAAGGAGTAGGAGAAACAGGGCTACCTTGGTTGGTGCGTGTACAGGATGAATTGGATATGCTTGCTACCACGGAAGTGGCTACTCGCGAACACGTAGAGCAAGCCATGCAAGCCGGTATCAGAATACTCTGGTTAGGTGCACGAACCACATCCAATCCCTTTGCTGTACAAGAAATTGCCGATACGATAGGCAAGGACGAATCGGTGATTGTCCTCGTCAAGAATCCGATCAGTCCCGATTTGGATCTGTGGACAGGAGCCCTAGAACGGCTTCGGCAGTCCGGAGTTCGACAGATCGGAGCCATCCATAGAGGATTCAGTACCTATGCGACCAAGACGTTTCGCAATCCTCCACATTGGCAGATTCCCTTCGATTTGAAAAGACGTTTTCCTTCGTTAACCATCCTTTGTGATCCGAGTCATATTACGGGACAGAGAGATCGGATCGAATCCGTCAGCCAGCAAGCCATGGAAATGAATTTTGACGGGCTGATCATTGAGTCGCATTGCTGTCCGGATAAGGCTCTAAGCGATGCAAGCCAGCAGATAACACCTACTGTACTTGCCCAAATCCTCCGGCGACTGCGTATCCCACGCCGCCAATCCGAAAAGCAGGACGCAGCGCTGATCTCTTGGCGCATGCAGATTGATCAGATAGATGAGAGTATAGTGGCATTGCTAGCTGCGCGGATGCAAGTGGCATACGAGATAGGTTTGTTCAAAAAAGAGCACAATCTGGCTGTGGTTCAGAATCTCCGCTACGAACAACTACAGCGCAACCGTGCCCGTACTGCAGCCCTCTTAGGTTTGGACGAAACATTTATATCGGAGCTATTCAGCCGTATTCATGAGGAATCTGTCCGTCTGCAGACCCTTGCCCCCCAAAAGCCACACACCGACGACTGTATATCATGA
